# Supplementary material for: Axonal defasciculation is restricted to specific branching points during regeneration of the lateral line nerve in zebrafish
Source: Development. 2026 Jan 8;153(16):dev205054. doi: 10.1242/dev.205054 (PMC12848573; doi:10.1242/dev.205054)
Supplement: Supplementary information [file develop-153-205054-s1.pdf]

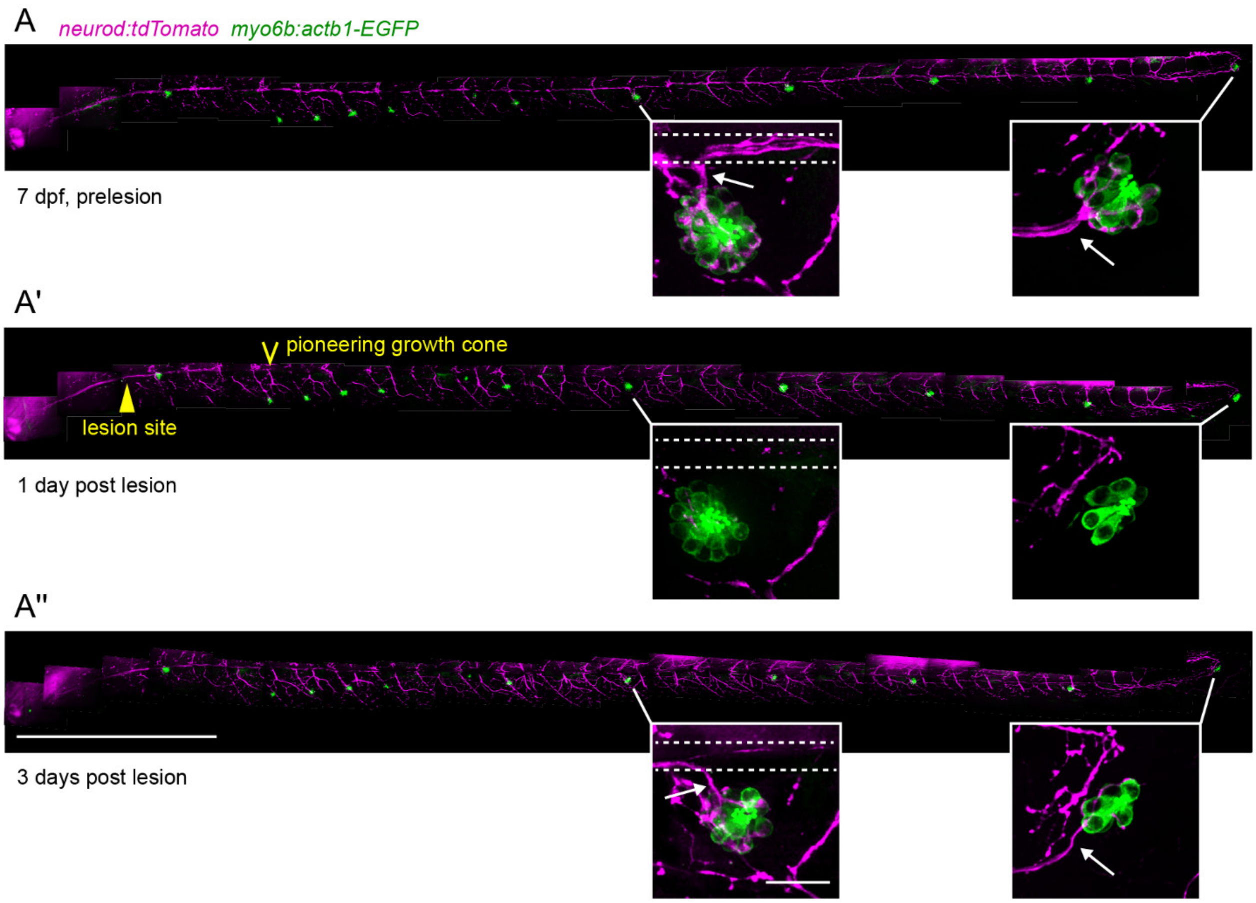

Fig. S1. Hair cells post nerve lesion.

(A) A representative 7 dpf *Tg(neurod:tdTomato, myo6b:actb1-EGFP)* larva before, 1 dpl **(A')**, and 3 dpl **(A'')**. *Neurod* is a pan-neuronal promoter driving expression of tdTomato (magenta) and the *myo6b* promoter selectively labels hair cells with GFP (green). At 1 dpl, leader growth cones (yellow caret) had partially extended along the horizontal myoseptum. The majority of pLL hair cells were distal to the cut axons and remained denervated (insets). At 3 dpl, axons have regenerated to the tail end of the larva and all the neuromasts have been reinnervated. Dotted white lines demarcate the path of the pLL nerve and the white arrows denotes axon defasciculation towards neuromasts. Scale bar: 500 μm; inset scale bar: 20 μm.

Development • Supplementary information

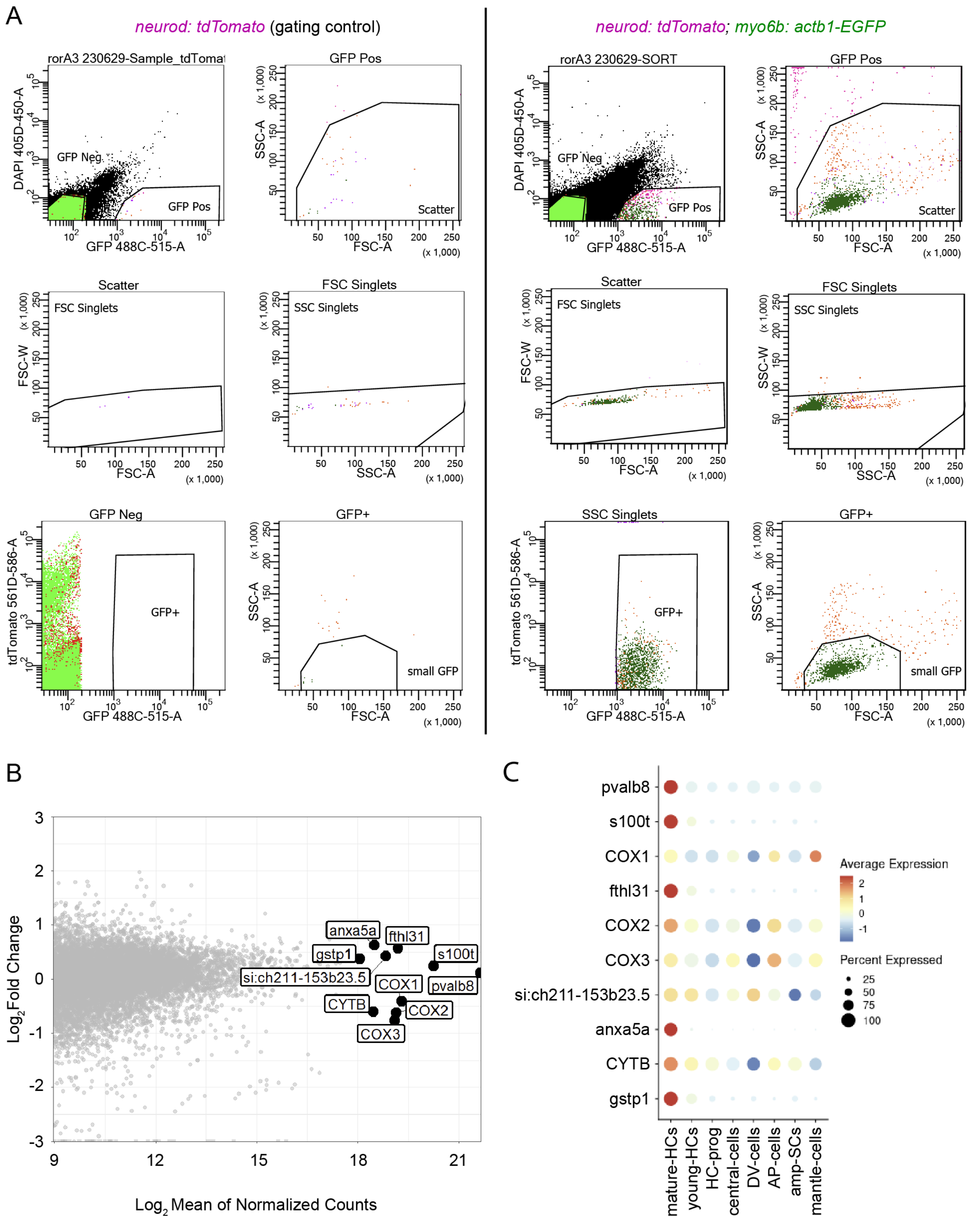

Fig. S2. Isolation of hair cells for bulk sequencing

(A) The gating strategy used for fluorescence-activated cell sorting (FACS) of GFP-positive hair cells in *Tg(neurod:tdTomato, myo6b:actb1-EGFP)* larvae following nerve

Development • Supplementary information

lesion (right). Singly transgenic *Tg(neurod:tdTomato)* larvae were used as gating controls for each session (left). FSC – forward scatter, SSC – side scatter. **(B)** An MA plot of transcripts sequenced in bulk hair cell samples 1 dpl compared to controls. The 10 most-expressed genes across all sample types are outlined in black. **(C)**The same genes in (B) cross-referenced to a published single-cell RNAseq dataset of the neuromast (Baek et al., 2022). The genes are selectively and highly expressed in hair cells, confirming the accuracy of the isolation and sorting protocol.

Development • Supplementary information

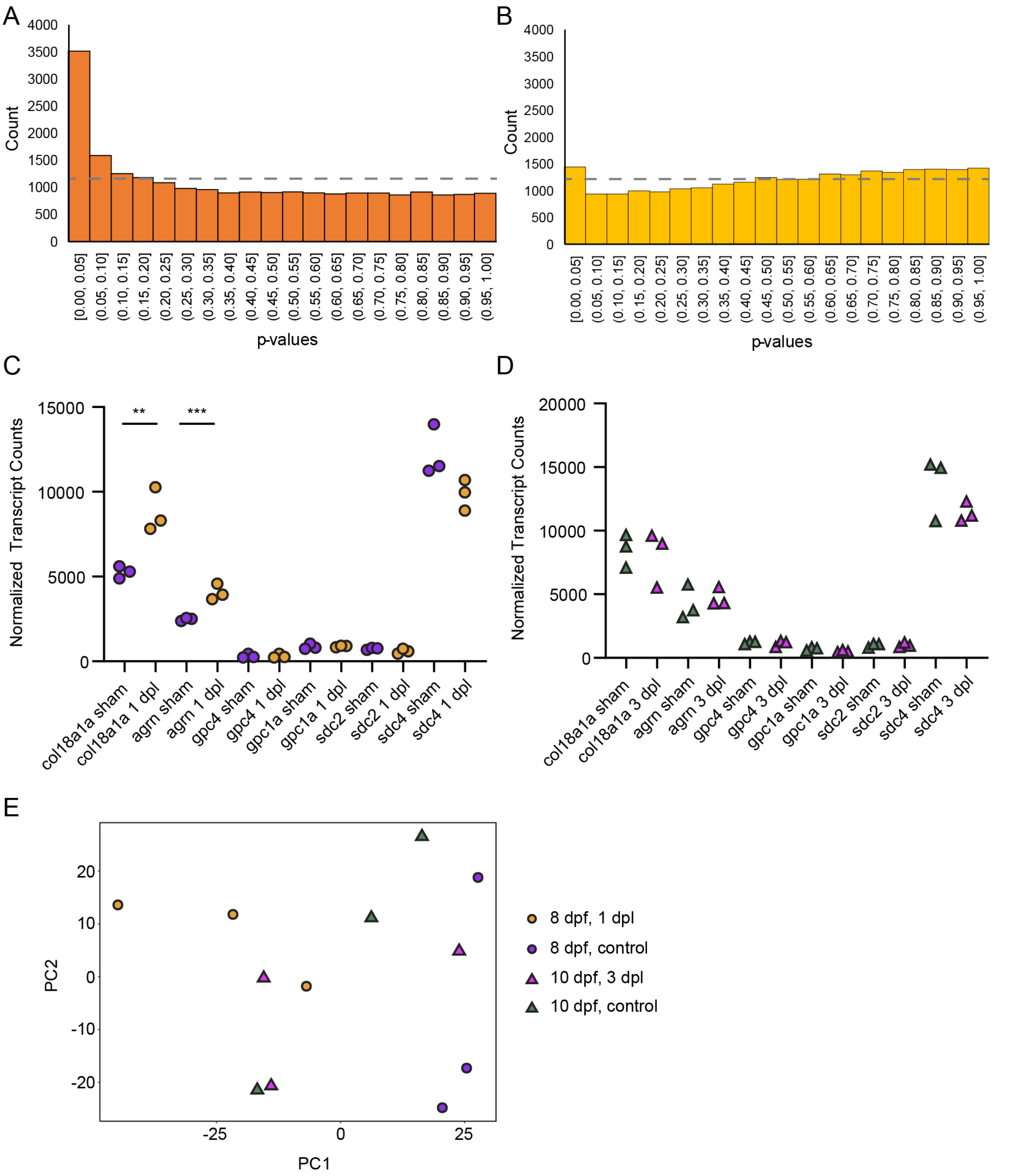

Fig. S3. Distribution of p-values from bulk sequencing data

(A) The distribution of *p-values* from testing the differential expression of each gene in hair cells 1 dpl compared to controls. A left-sided peak of values below 0.05 deviates from a uniform distribution (gray dotted line). n = 22,148 total genes tested. **(B)** The distribution of *p-values* from testing the differential expression of each gene in hair cells 3 dpl

Development • Supplementary information

compared to controls. The distribution more closely resembles a uniform distribution compared to (A). n = 24,209 total genes tested. **(C)** The normalized transcript counts from each sequenced replicate for the genes coding for secreted and transmembrane bound HSPGs in hair cell samples 1 dpl (orange) compared to age-matched controls (purple) and **(D)** hair cell samples 3 dpl (magenta) compared to age-matched controls (magenta). **(E)** A batch-corrected principal component analysis plot of all sequenced samples. There is greater distance between hair cells 1 dpl and their age-matched innervated controls than between hair cells 3 dpl and their age-matched innervated controls. PC – principal component. ** adjusted *p*-value < 0.01, *** adjusted *p*-value <0.001, Wald test.

Development • Supplementary information

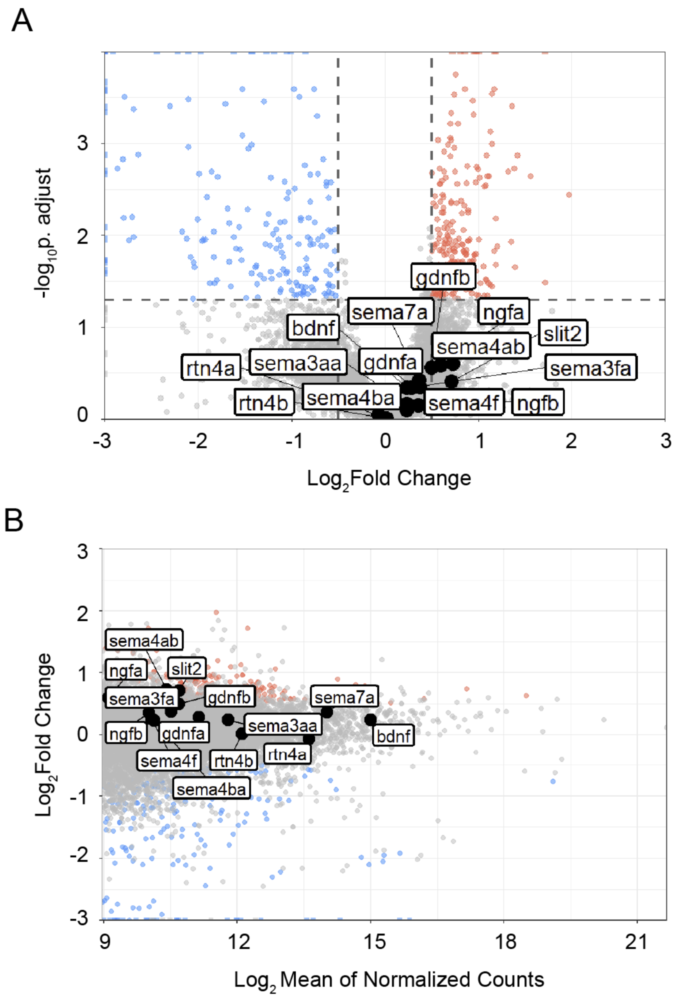

Fig. S4. Expression of axon guidance cues in denervated hair cells

(A) A volcano plot showing canonical axon guidance cues are not differentially expressed in hair cells 1 dpl compared to innervated controls. Statistical significance set at a *p-value* of 0.05 adjusted for multiple comparisons (horizontal dashed line) and biological significance set at 0.5 $\log_2$fold change (vertical dashed line). **(B)** An MA plot of genes sequenced in hair cells 1 dpl compared to control with canonical axon guidance cues highlighted in black.

Development • Supplementary information

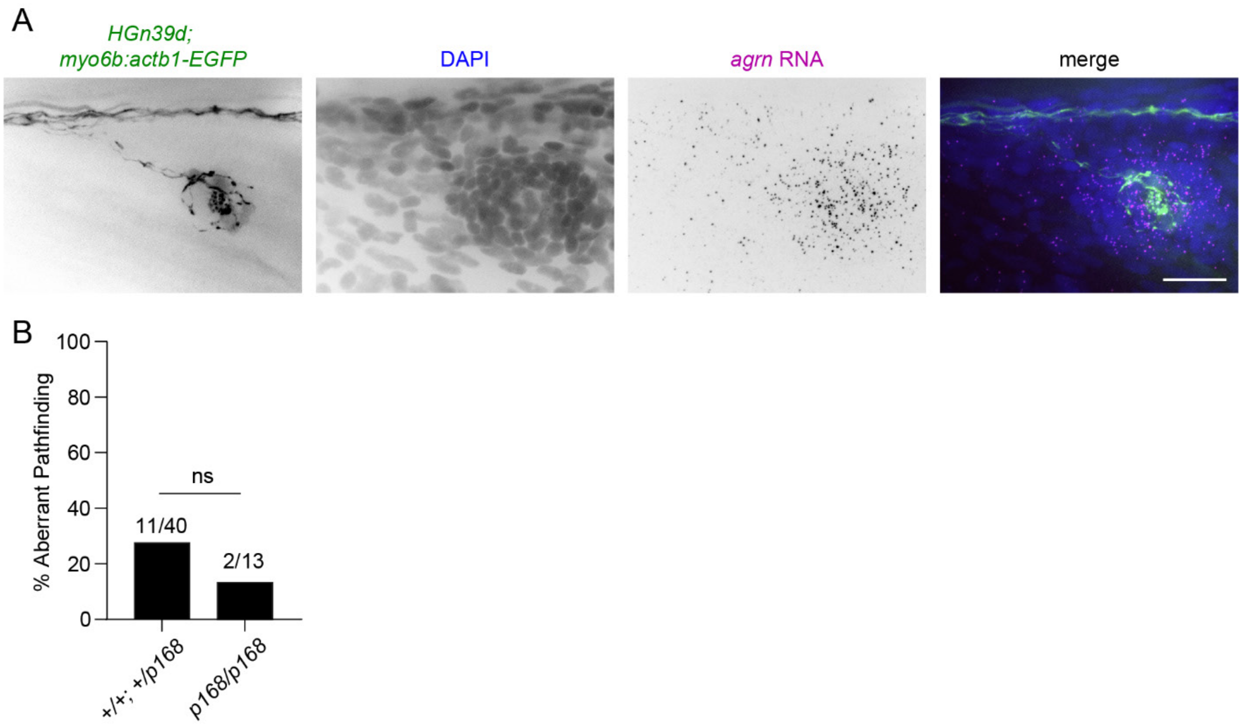

Fig. S5. Normal nerve regeneration in *agrn*^{p168} mutants

(A) RNA-FISH of *agrn* in a fixed 8 dpf *Tg(HGn39d; myo6b:actb1-EGFP)* larva. Expression of *agrn* is specific to hair cells and supporting cells of the neuromast. **(B)** There is an absence of aberrant afferent axon pathfinding during nerve regeneration in *agrn*$^{p168/p168}$ *Tg(HGn39d; myo6b:actb1-EGFP)* mutants compared to siblings. n = 40 *+/+* and *+/p168* siblings, n = 13 *p168/p168* mutants. ns – not significant, Fisher's exact test. Scale bar: 20 µm.

Development • Supplementary information

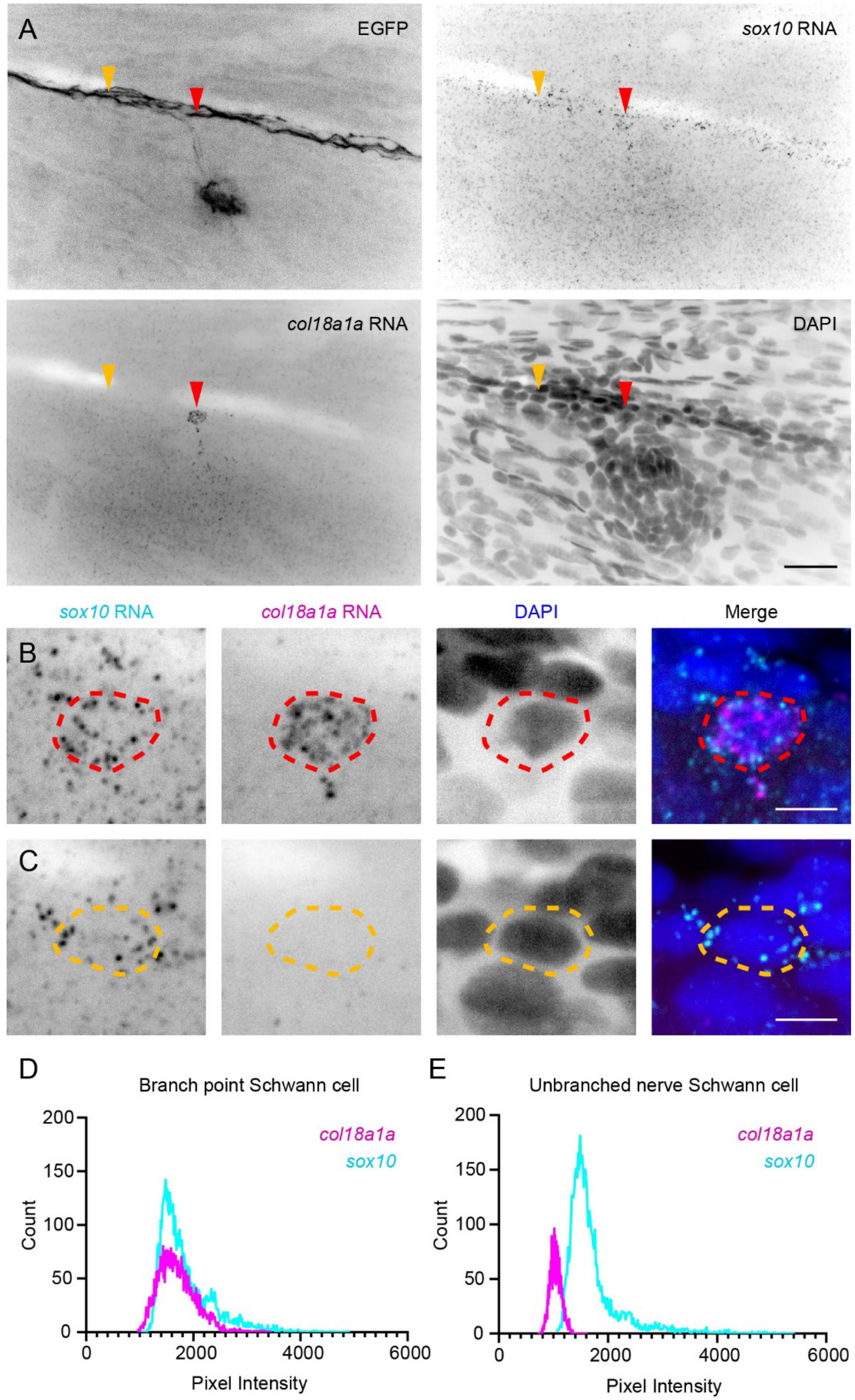

Development • Supplementary information

Fig. S6. Colocalization of *sox10* and *col18a1a* RNA only at defasciculation points
(A) Maximum projection images of individual color channels from RNA-FISH of *col18a1a* and *sox10* in a fixed 8 dpf *Tg(HGn39d; myo6b:actb1-EGFP)* larva. A Schwann cell at the axonal branching point is positive for *col18a1a* (red arrowhead) and a Schwann cell lining an unbranched portion of the nerve (orange arrowhead) is negative for *col18a1a*. **(B, C)** Individual color channels from a single confocal plane from the Schwann cells marked in (A) (red arrowhead – B, yellow arrowhead – C), and an overlayed image on the right. The DAPI channel was used to associate probe signal with individual cells. **(D, E)** A histogram of the pixel intensities associated with *col18a1a* and *sox10* probe signals in the ROIs defined in (B) and (C) around Schwann cell nuclei. ROI – Region of Interest. Scale bar: 20 μm in A, 5 μm in B, C.

Development • Supplementary information

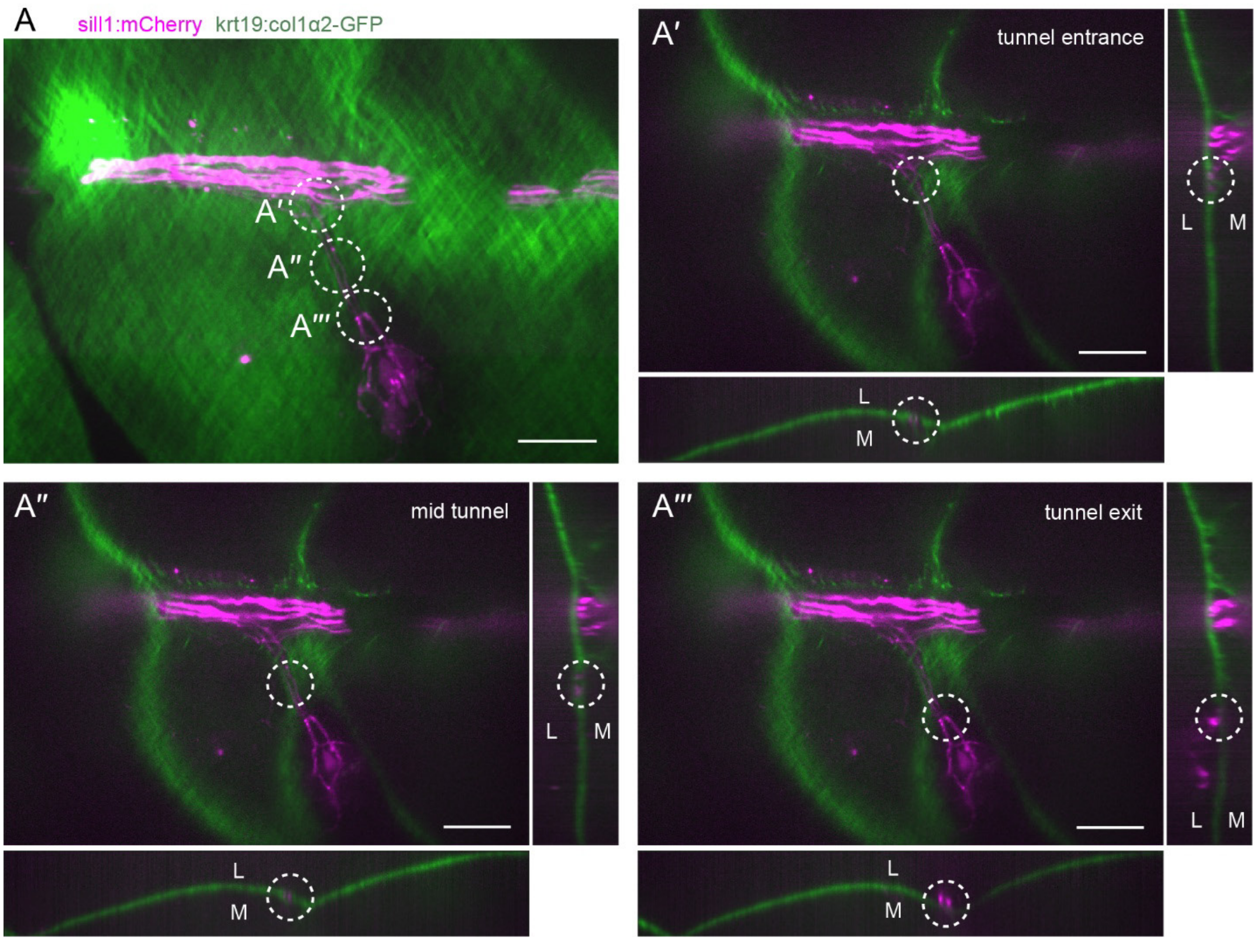

Fig. S7. Innervation of secondary neuromasts through collagen tunnel (A) A maximum projection image of a confocal stack capturing the innervation of a secondary neuromast in a 5 dpf *Tg(krt19:col1α2-GFP, sill1:mCherry)* larva. **(A')** Orthogonal planes of the confocal stack at the location of axon defasciculation, which is just medial to the entrance of the collagen I matrix tunnel. **(A')** Orthogonal planes of the confocal stack at the midpoint of the collagen I matrix tunnel. The defasciculated axons are embedded within the matrix itself. **(A'')** Orthogonal planes of the confocal stack at the exit of the collagen I matrix tunnel at the secondary neuromast, which resides lateral to the epidermal boundary layer. Dotted white outlines demarcate the defasciculated axons in each imaging plane. For (A'-A'''), Center pane – sagittal plane, right pane – axial plane, bottom pane – coronal plane. L – lateral, M – medial. Scale bar: 20 μm.

Development • Supplementary information

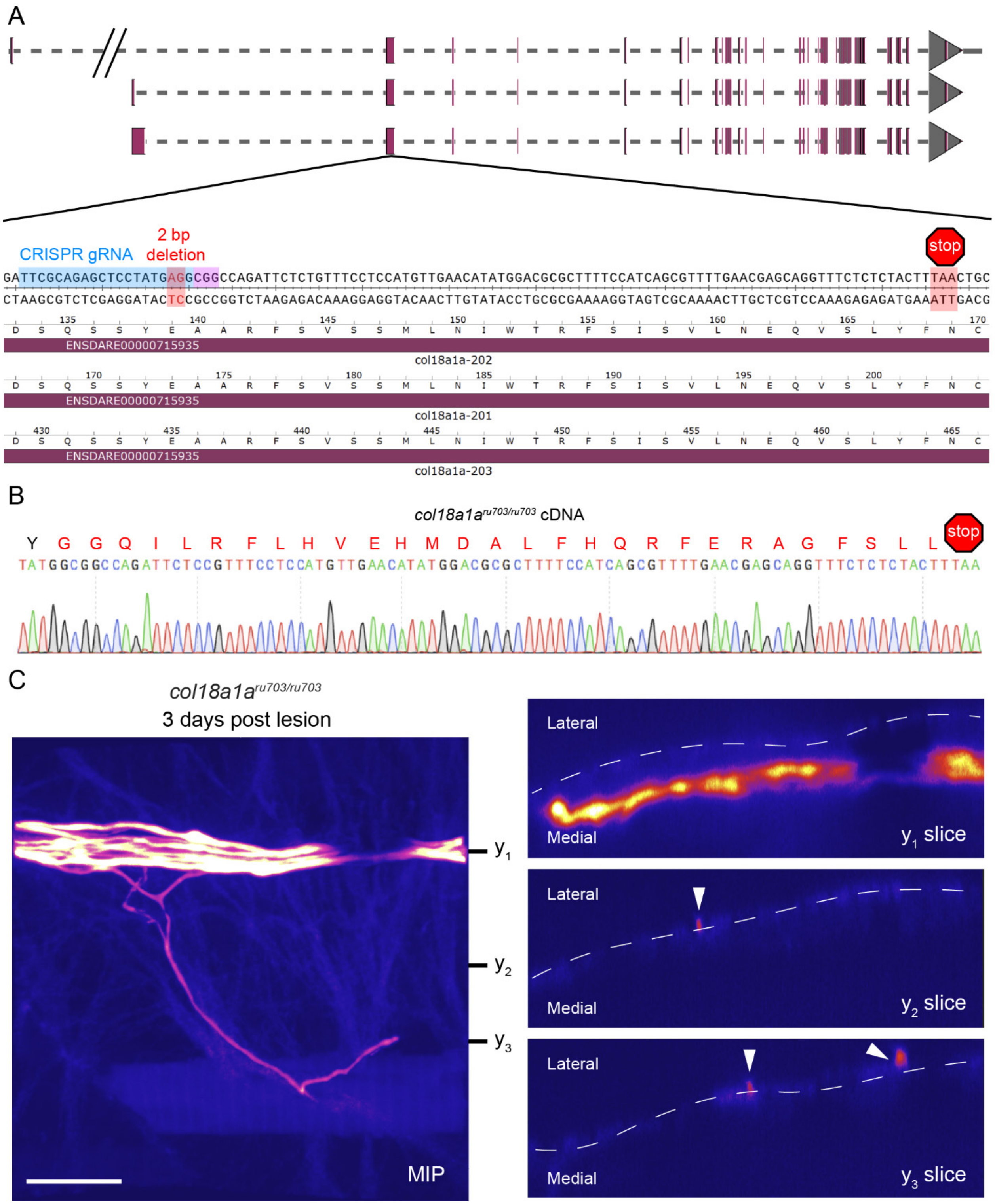

Fig. S8. *col18a1a*^{ru703} mutation

(A) The CRISPR gRNA sequence (highlighted in blue) with adjacent CGG Cas9 PAM motif (pink) used to generate the *ru703* mutant line. The gRNA is complementary to either

Development • Supplementary information

exon 2 in the 201 and 203 isoforms of *col18a1a*, and exon 3 in the 202 isoform. The two base pair AG deletion in *ru703* mutants are marked in red. A premature stop codon is created downstream of the frameshift mutation. **(B)** A chromatogram of sequenced cDNA reverse transcribed from total RNA isolated from homozygous mutant *col18a1a*$^{ru703/ru703}$ larvae. Mutant codons and the premature stop codon marked in red. **(C)** A maximum projection image (MIP, left) of an aberrant axon branch in an 8 dpf *Tg(krt19:col1α2-GFP; HGn39d; myo6b:actb1-EGFP) col18a1a*$^{ru703/ru703}$ mutant 3 days following nerve lesion, and coronal slices (right) taken at the planes indicated. The aberrant branch (white arrowhead) crosses the epidermal boundary layer (dotted white outline) and extends on the lateral side. Scale bar: 10 μm in C.

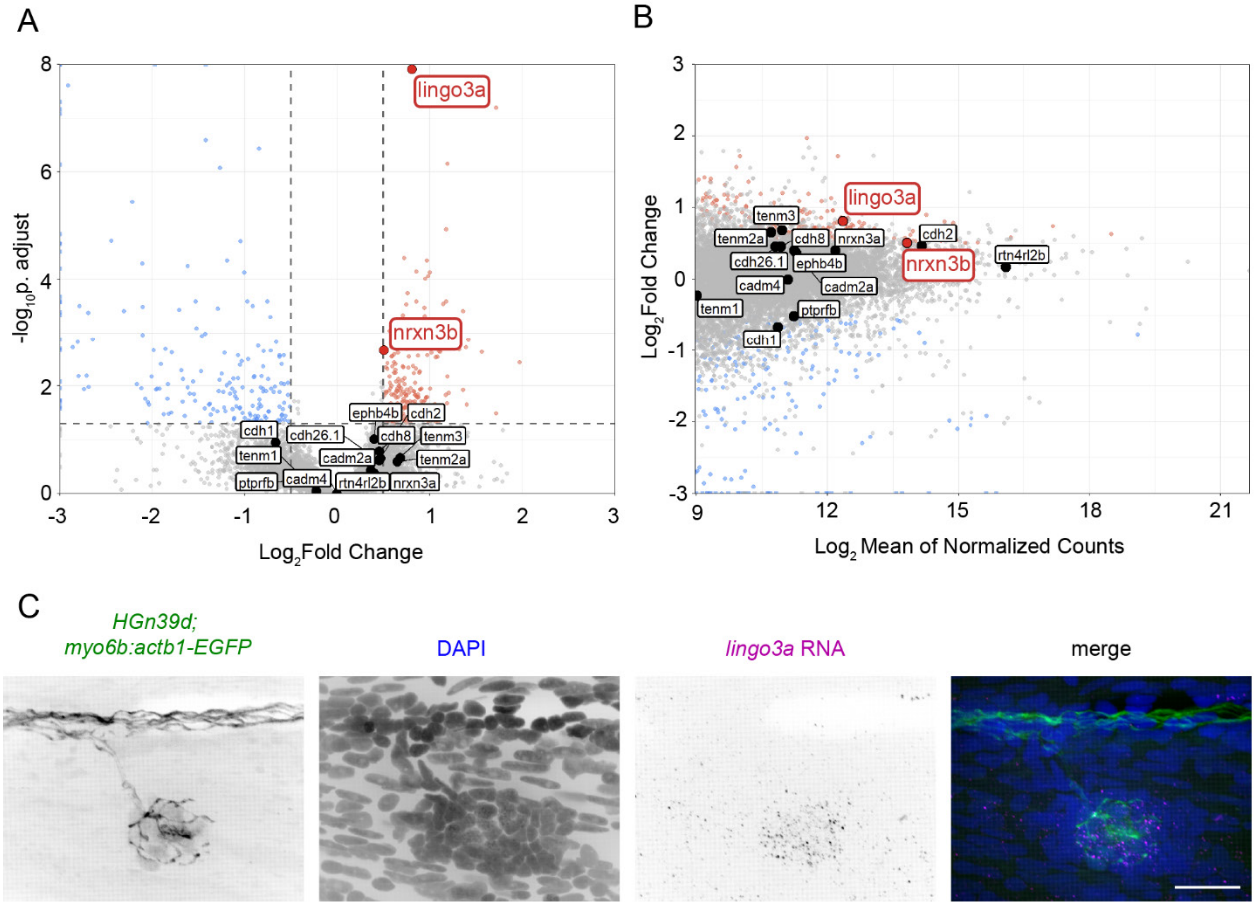

Fig. S9. Synaptic adhesion genes upregulated in denervated hair cells (A) A volcano plot with canonical synaptic adhesion molecule genes highlighted. *Lingo3a* and *nrxn3b* are upregulated (red) in denervated hair cells 1 dpl compared to innervated controls. Statistical significance set at a p-value of 0.05 adjusted for multiple comparisons (horizontal dashed line) and biological significance set at 0.05 $\log_2$fold change (vertical dashed line). **(B)** An MA plot of genes sequenced in hair cells 1 dpl compared to control with canonical synaptic adhesion molecule genes highlighted. **(C)** RNA-FISH against *lingo3a* in an 8 dpf *Tg(HGn39d; myo6b:actb1-EGFP)* larva reveals restricted expression in only hair cells of the neuromast. Scale bar: 20 µm.

Development • Supplementary information

Table S1. Spreadsheet of differential gene expression in hair cells one day post nerve lesion

Available for download at
https://journals.biologists.com/dev/article-lookup/doi/10.1242/dev.205054#supplementary-data

Table S2. Spreadsheet of differential gene expression in hair cells three days post nerve lesion

Available for download at
https://journals.biologists.com/dev/article-lookup/doi/10.1242/dev.205054#supplementary-data

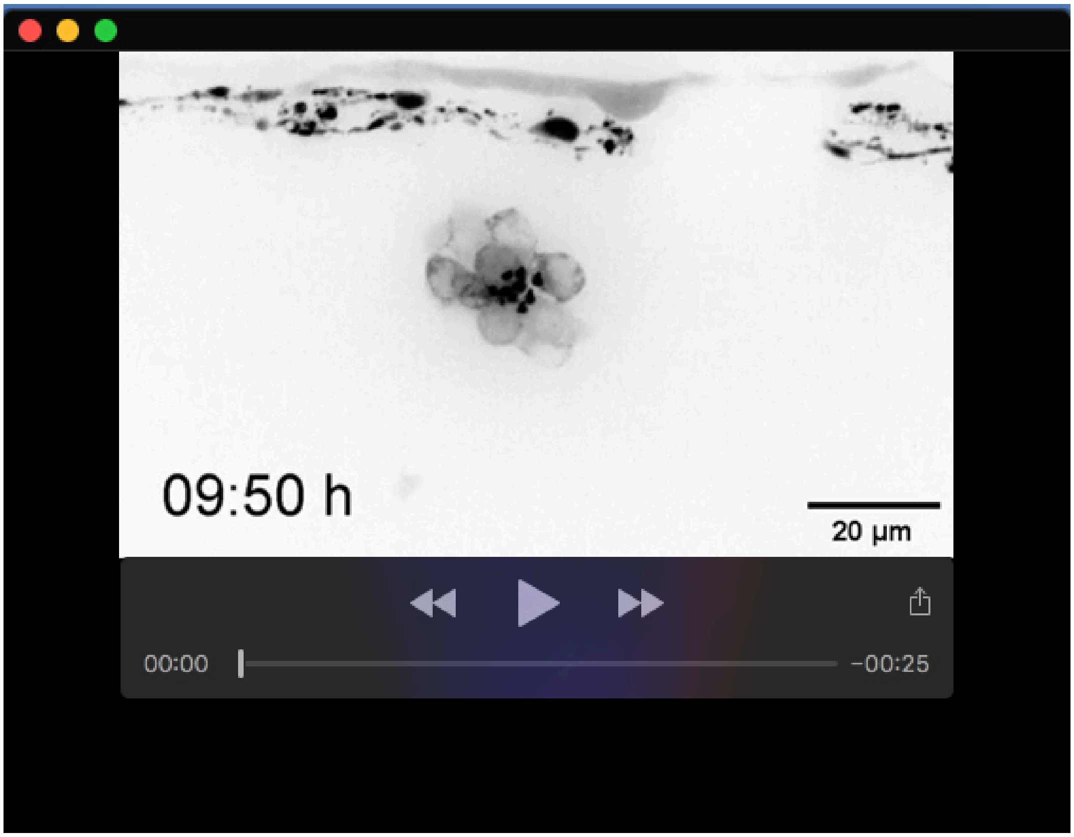

Movie 1. Neuromast reinnervation
A timelapse of nerve regeneration and neuromast reinnervation following nerve lesion in a 4 dpf *Tg(myo6b:actb1-EGFP, HGn39d)* larva.

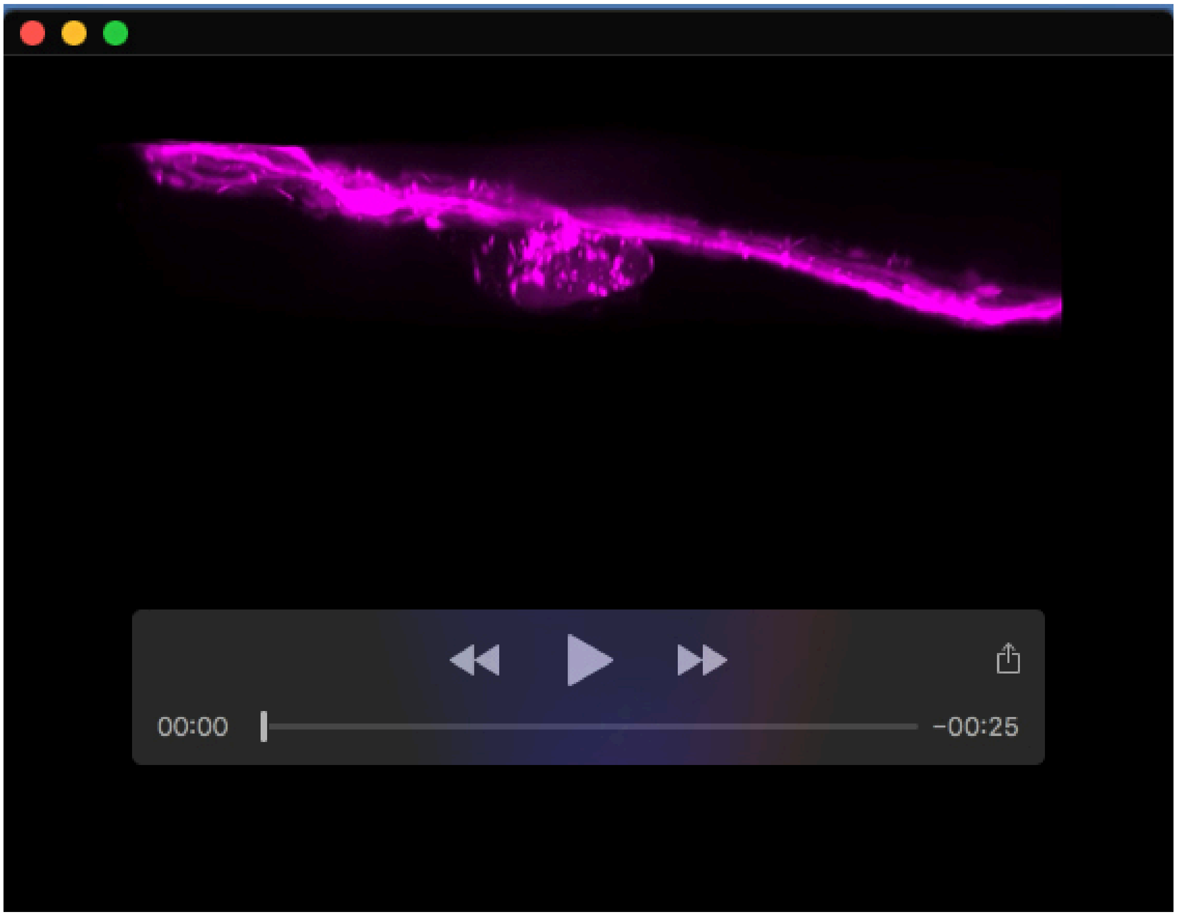

Movie 2. Axon defasciculation through gap in the extracellular matrix

A confocal imaging stack viewed in three dimensions depicting the defasciculation of lateral line afferents (magenta) through a discrete gap in the collagen I matrix (green) that separates the epidermis from the underlying subepidermal space where the nerve is located.

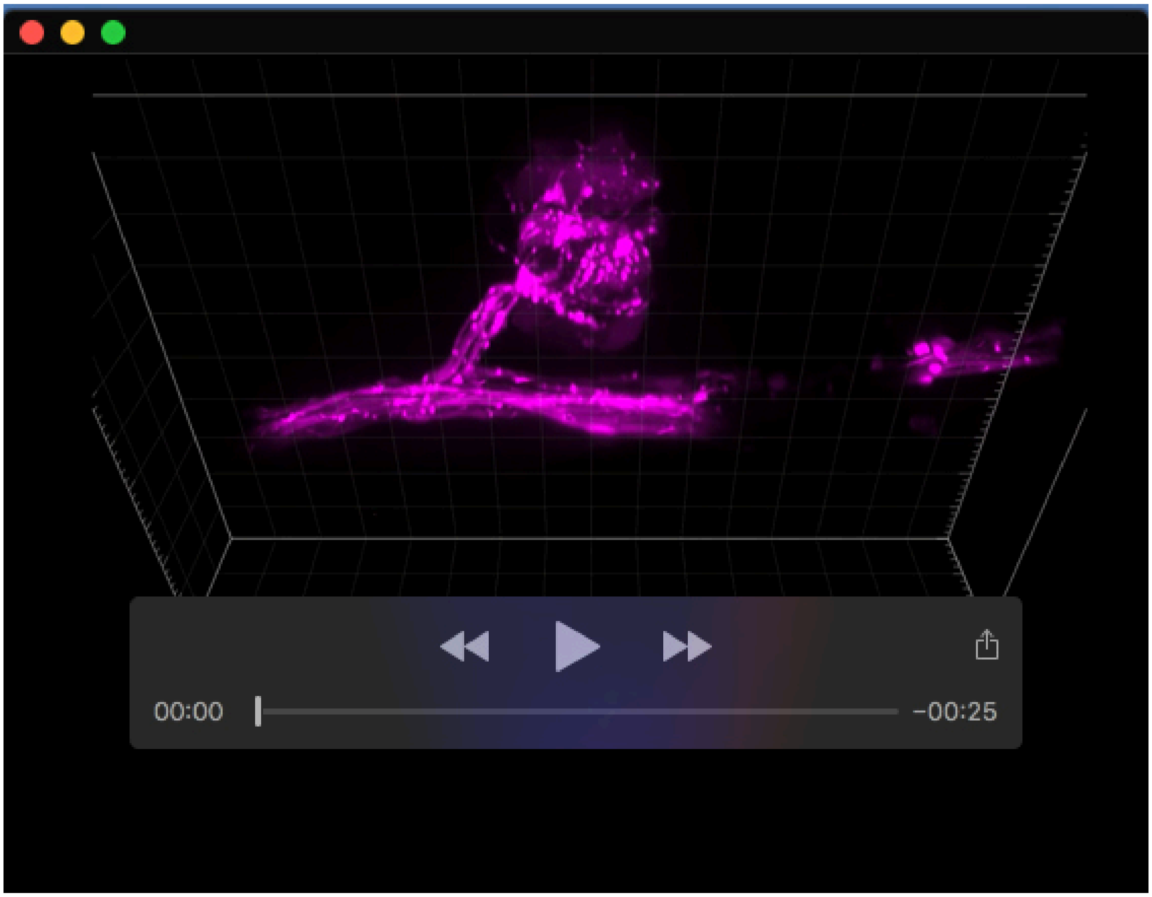

Movie 3. Selective defasciculation through native gap following nerve lesion

A confocal imaging stack viewed in three dimensions depicting a regenerated nerve 3 days post nerve lesion (magenta) with individual axons selectively defasciculating through the native gap in the extracellular matrix rather than through an artificially made gap.
